# miR‐15b modulates multidrug resistance in human osteosarcoma *in vitro* and *in vivo*

**DOI:** 10.1002/1878-0261.12015

**Published:** 2016-10-24

**Authors:** Zhenfeng Duan, Yan Gao, Jacson Shen, Edwin Choy, Gregory Cote, David Harmon, Karen Bernstein, Santiago Lozano‐Calderon, Henry Mankin, Francis J. Hornicek

**Affiliations:** ^1^ Sarcoma Biology Laboratory Center for Sarcoma and Connective Tissue Oncology Massachusetts General Hospital Boston MA USA

**Keywords:** doxorubicin, miR‐15b, multidrug resistance, osteosarcoma, Wee1

## Abstract

The development of multidrug resistance (MDR) in cancer cells to chemotherapy drugs continues to be a major clinical problem. MicroRNAs (miRNA, miR) play an important role in regulating tumour cell growth and survival; however, the role of miRs in the development of drug resistance in osteosarcoma cells is largely uncharacterized. We sought to identify and characterize human miRs that act as key regulators of MDR in osteosarcoma. We utilized a miR microarray to screen for differentially expressed miRs in osteosarcoma MDR cell lines. We determined the mechanisms of the deregulation of expression of miR‐15b in osteosarcoma MDR cell lines, and its association with clinically obtained tumour samples was examined in tissue microarray (TMA). The significance of miR‐15b in reversing drug resistance was evaluated in a mouse xenograft model of MDR osteosarcoma. We identified miR‐15b as being significantly (*P* < 0.01) downregulated in KHOS_MR_ and U‐2OS_MR_ cell lines as compared with their parental cell lines. We found that Wee1 is a target gene of miR‐15b and observed that transfection with miR‐15b inhibits Wee1 expression and partially reverses MDR in osteosarcoma cell lines. Systemic *in vivo* administration of miR‐15b mimics sensitizes resistant cells to doxorubicin and induces cell death in MDR models of osteosarcoma. Clinically, reduced miR‐15b expression was associated with poor patient survival. Osteosarcoma patients with low miR‐15b expression levels had significantly shorter survival times than patients with high expression levels of miR‐15b. These results collectively indicate that MDR in osteosarcoma is associated with downregulation of miR‐15b, and miR‐15b reconstitution can reverse chemotherapy resistance in osteosarcoma.

**Abbreviations**
MDR: multidrug resistance
miR‐15b: microRNA‐15b

## 1 Introduction

Osteosarcoma is the most common primary bone cancer in children and remains a leading cause of cancer death in adolescents. Standard treatment for osteosarcoma is surgery and chemotherapy, but treatment paradigms and survival rates have not improved in almost three decades (Gill *et al*., 2013; Isakoff *et al*., 2015). Drug treatment usually includes a combination of doxorubicin, methotrexate and cisplatin. Unfortunately, the efficacy of these agents is hampered by the eventual development of multidrug resistance (MDR) (Hattinger *et al*., 2015; Yang *et al*., 2014). The mechanism of acquiring MDR in sarcoma is not well understood. Several potential mechanisms, including overexpression of the MDR1 (ABCB1) gene, which encodes the ABC drug efflux pump P‐glycoprotein (Pgp), and elevation of the apoptotic threshold that contributes to drug resistance, have been proposed to play important roles for tumour cells acquiring MDR. However, the contribution of these mechanisms to clinical resistance remains controversial (Duan *et al*., 2005; Hattinger *et al*., 2015; Isakoff *et al*., 2015). Strategies for reversing MDR by targeting Pgp have been studied extensively in different MDR model systems and in clinical trials, including for osteosarcoma, but have been met with limited results. Reversing drug resistance is still one of the biggest impediments to successfully treating osteosarcoma. Recently, experimental evidence from several laboratories has implicated that deregulation of miRs (also known as microRNAs or miRNAs) may contribute to chemotherapy drug resistance (Allen and Weiss, 2010; Holleman *et al*., 2011; Jones *et al*., 2012; Kobayashi *et al*., 2012; Kovalchuk *et al*., 2008; Zheng *et al*., 2010).

miRs are a subset of small noncoding RNA molecules that influence tumour formation, maintenance, metastasis, apoptosis and drug resistance (Croce, 2009; Kobayashi *et al*., 2012; Kong *et al*., 2012; Sampson *et al*., 2015; Tong and Nemunaitis, 2008). Mature miRs bind to the 3′‐untranslated regions (UTRs) of target genes and inhibit gene expression by degradation or repressing translation of the target mRNA. More than 1880 annotated human miRs have been registered at miRBase (release 21.0, http://microrna.sanger.ac.uk/). There has been a great interest in the function of miRs in human cancers, including in osteosarcoma. Numerous studies have observed dysregulation of miRs in tumours by microarray assay or by RNA sequencing (Duan *et al*., 2010, 2011; Kobayashi *et al*., 2012). Several studies have demonstrated the involvement of miRs in the pathogenesis of osteosarcoma with the potential for development in disease diagnostics and therapeutics (Kobayashi *et al*., 2012; Sampson *et al*., 2015; Zheng *et al*., 2010). Aberrant expression of miRs in osteosarcoma is mainly divided into two classes: upregulated miRs and downregulated miRs. Each individual miR can affect almost every aspect of tumour cell behaviour, including the regulation of cellular development, differentiation and proliferation.

A number of research findings, both laboratory‐ and clinic‐based, have reported on the implications of miRs in chemoresistance (Croce, 2009; Gill *et al*., 2013; Kobayashi *et al*., 2012; Ma *et al*., 2010). Several research groups have shown that the expressions of miRs in chemoresistant cancer cells and their parental chemosensitive ones are significantly different. The significant correlation between miR expression patterns and compound potency in the NCI‐60 cancer cell lines panel suggests that miRs have a role in chemoresistance (Blower *et al*., 2007). Several miRs have been shown to sensitize cancer cells that have acquired MDR to chemotherapy drugs by downregulating miR‐targeted genes (Kobayashi *et al*., 2012; Kovalchuk *et al*., 2008; Sampson *et al*., 2015; Wang *et al*., 2015). Although these studies suggest that miRs have important roles in the development of chemosensitivity or chemoresistance in different cancers, including breast, lung, colon, stomach, liver, ovarian and prostate cancer, the potential role of miRs in the development of drug resistance in osteosarcoma is largely uncharacterized.

To identify the miRs that are important in the development of drug resistance in osteosarcoma cells, in this study, we have utilized miR arrays to identify miRs associated with the development of MDR in two pairs of osteosarcoma MDR cell lines. Subsequently, we evaluated the mechanisms of the deregulation of expression of the most significantly altered miR (miR‐15b) in osteosarcoma MDR cell lines and identified Wee1 as a miR‐15b‐targeted gene. Wee1 is a protein kinase that regulates the G2 checkpoint in response to DNA damage. Overexpression of Wee1 has been shown to be correlated with poor outcomes, recurrence and drug resistance in various cancers (Do *et al*., 2013; Hamilton *et al*., 2014; Kreahling *et al*., 2013). However, the association and biological functions of miR‐15b and Wee1 have not yet been investigated in osteosarcoma. We further examined the expression of miR‐15b and Wee1 in a tissue microarray (TMA) panel of clinical osteosarcoma samples. The *in vivo* significance of miR‐15b in reversing drug resistance was evaluated in a mouse xenograft model of MDR osteosarcoma. Finally, we correlated the reduced miR‐15b expression with poor patient survival in patients with osteosarcoma.

## 2 Materials and methods

### 2.1 Human osteosarcoma cell line culture

The human osteosarcoma cell line KHOS and the drug‐resistant osteosarcoma cell line KHOS_MR_ were kindly provided by Dr. Efstathios Gonos (Institute of Biological Research & Biotechnology, Athens, Greece) (Lourda *et al*., 2007). The human osteosarcoma cell line U‐2OS was purchased from the ATCC, and its drug‐resistant cell line U‐2OS_MR_ was generated as previously reported (Yang *et al*., 2009a,b). The human ovarian cancer cell line SKOV‐3 and the paclitaxel‐resistant variant SKOV‐3_TR_, the human breast cancer cell line MCF‐7 and the paclitaxel‐resistant variant MCF‐7_TR_ have been described previously (Duan *et al*., 2005, 2008). These osteosarcoma, ovarian cancer and breast cancer drug‐resistant cell lines have been characterized with the stable MDR phenotype in previous studies. All of the cell lines were cultured in RPMI 1640 (Life Technologies‎, Carlsbad, CA, USA) supplemented with 10% FBS, 100 U·mL^−1^ penicillin and 100 μg·mL^−1^ streptomycin (Life Technologies). Cells were incubated at 37 °C in a humidified atmosphere, with 5% CO_2_–95% air and passaged twice every 2–3 days.

### 2.2 Human osteosarcoma tissue samples

Frozen tissue samples from 49 patients who had undergone surgical resection of osteosarcoma were obtained from the Massachusetts General Hospital (MGH) sarcoma tissue bank and were studied according to the policies of the institutional review board of the hospital. This study was conducted with the approval of the MGH Institutional Review Board (IRB protocol #:2007‐P‐002464/5). All specimens were assessed by light microscopy and immunohistochemistry.

### 2.3 Isolation of miRs and miR microarray assay

Total miRs were extracted from cancer cell lines using the miRNANeasy Mini Kit (Qiagen GmbH, Hilden, Germany) by following the manufacturer's instructions. A similar protocol was used to isolate miRs from the osteosarcoma frozen tissues. The purity and quantity of the isolated small miRs were assessed based on the quality of total RNA using 1% formaldehyde–agarose gel electrophoresis and by spectrophotometer measurement (Beckman, Columbia, MD, USA). The miR samples were submitted to LC Sciences (Houston, TX, USA) for further analysis by Agilent Bioanalyzer. The miR microarray assay was performed using a service provider (LC Sciences). The assay started from a 5‐μg total RNA sample, which was size‐fractionated using a YM‐100 Microcon centrifugal filter (Millipore, Billerica, MA, USA), and the small RNAs (< 300 nucleotides) isolated were 3′‐extended with a poly(A) tail using poly(A) polymerase. An oligonucleotide tag was then ligated to the poly(A) tail for later fluorescent dye staining; two different tags were used for the two RNA samples in dual‐sample experiments. Hybridization was performed overnight on a μParaflo microfluidic chip (miRHuman_16.0) using a microcirculation pump (Atactic Technologies, Houston, TX, USA). On the microfluidic chip, each detection probe consisted of a chemically modified nucleotide coding segment complementary to target miR (from miRNABase, http://microrna.sanger.ac.uk/sequences/) or other RNA (control sequences). The hybridization melting temperatures were balanced by chemical modifications of the detection probes. Hybridization used 100 μL 6× SSPE buffer (0.90 m NaCl, 60 mm Na_2_HPO_4_, 6 mm EDTA, pH 6.8) containing 25% formamide at 34 °C. After RNA hybridization, tag‐conjugating Cy3 and Cy5 dyes were circulated through the microfluidic chip for dye staining. Fluorescence images were collected using a laser scanner (GenePix 4000B; Molecular Devices, Sunnyvale, CA, USA) and digitized using array‐pro image analysis software (Media Cybernetics, Silver Spring, MD, USA).

### 2.4 Hierarchical cluster analysis of miR expression and statistical analysis

Multiple‐sample analysis involves normalization, data adjustment, *t*‐test and clustering. Normalization was carried out using a cyclic LOWESS (locally weighted regression) method. The normalization removed system‐related variations, such as sample amount variations, different labelling dyes and signal gain differences of scanners so that biological variations could be accurately detected. Intensity values were log2‐transformed for further analysis. Gene centring and normalization transformed the log2 values using the mean and the SD of individual genes across all samples using the following formula: Value = [(Value) – Mean (Gene)]/[SD (Gene)]. For hierarchical cluster analysis, the clustering was performed using a hierarchical method and with an average linkage and Euclidean distance metric. All data processes, except the clustering plot, were carried out using in‐house (LC Sciences)‐developed computer programs. The clustering plot was generated using tigr mev (Multiple Experimental Viewer) software from The Institute for Genomic Research. For statistical analysis of microarray data, a *t*‐test was performed between the ‘control’ and ‘test’ sample groups. *t*‐values were calculated for each miR, and *P*‐values were computed from the theoretical *t*‐distribution. miRs with *P*‐values below a critical *P*‐value (typically 0.01) were selected for cluster analysis.

### 2.5 Validation and quantification of miR expression by miR TaqMan reverse transcription PCR

Real‐time reverse transcription PCR (RT‐PCR) was performed to validate differentially expressed miRs in osteosarcoma cell lines. Expressions of miR‐15b were determined in osteosarcoma tissues. For mature miR detection, cDNA reverse transcription was performed from total RNA samples using specific miR primers from the TaqMan miR Assay and reagents from the TaqMan miR Reverse Transcription kit (Life Technologies, Foster City, CA, USA). The resulting cDNA was amplified by PCR using TaqMan miR Assay primers with the TaqMan Universal PCR Master Mix and analysed with a StepOnePlus Real‐Time PCR System (Life Technologies) according to the manufacturer's instructions. Expression of RNU48 miR was used as an endogenous control. The relative levels of miR‐15b expression were calculated from the relevant signals by normalization with the signal for RNU48 miR expression. All reactions were performed in triplicate. The relationships between miR‐15b expression levels and clinical features of patients with osteosarcoma were analysed by chi‐square test and Student's *t*‐test. The miR‐15b expression levels were divided by the median value. The high‐expression group (> 600) was set above the median value, and the low‐expression group (≤ 600) was set below the median value. Overall survival curves were analysed with the Kaplan–Meier method and compared using the log‐rank test. A statistically significant difference was defined as *P*‐value less than 0.05.

### 2.6 Evaluation of miR‐15b copy number

To determine potential miR‐15b copy number variation between chemoresistance and their parental sensitive cell lines, the TaqMan Copy Number Assay (Applied Biosystems, Foster City, CA, USA) was performed according to the manufacturer's protocol. Briefly, 20 ng of genomic DNA was prepared in a final volume of 20 μL containing Reference Assay human RNase P (VIC^®^ dye‐labelled TAMRA™ probe) and Custom Assay miR‐15b (FAM^™^ dye‐labelled NFQ‐MGB probe; probe: 5′‐CTACAGTCAAGATGCGAATCAT‐3′; FWD: 5′‐CTGTAGCAGCACATCATGGTTTAC‐3′; REV: 5′‐GAATGAATTTCCTTAAATTTCTAGAGCAGCAA‐3′). The absolute quantification method was used to determine C_T_ data from the duplex PCR run on the StepOnePlus Real‐Time PCR System. The reaction was run as follows: 95 °C for 10 min, followed by 40 cycles of 95 °C for 15 s and 60 °C for 60 s. Afterwards, results were analysed on copycaller ^™^ Software v2.0 (Applied Biosystems) with Manual C_T_ threshold: 0.2; Autobaseline: On; and Predicted Copy Number: 2. Experiments were carried out with four replicates. Data were presented as copy number ± SD.

### 2.7 Transfection with miR‐15b precursor (miR‐15b mimics) and transfection with Wee1 siRNA

The human osteosarcoma KHOS_MR_ and U‐2OS_MR_ MDR cells were transfected with Lipofectamine™ RNAiMAX (Life Technologies) and pre‐miR‐15 precursor (hsa‐miR‐15b, Ambion^®^ Pre‐miR™ miR Precursor; Ambion^®^, Austin, TX, USA) or nonspecific control miR precursor according to the manufacturer's instructions. These miR‐15b precursor molecules (ready‐to‐use miR mimics) are small, chemically modified double‐stranded RNA molecules designed to mimic endogenous mature miR‐15b. They enable detailed study of miR‐15b biological effects via gain‐of‐function experiments. The sequence of the mature miR for pre‐miR‐15b was 5′‐UAGCAGCACAUCAUGGUUUACA‐3′.

Wee1 knockdown in osteosarcoma cells was performed by transfection with synthetic human Wee1 siRNA (GenBank Accession Number NM_003390, 5′‐CATGTAACAAGGATCTCCA‐3′) purchased from Sigma‐Aldrich (St. Louis, MO, USA). Nonspecific siRNA oligonucleotides were used as negative control. The siRNA oligonucleotides were dissolved in nuclease‐free water at a concentration of 100 mmol·L^−1^ and kept at −20 °C until the following transfection experiment. The effects of Wee1 siRNA on cellular growth and proliferation were assessed *in vitro* using the MTT assay. Experiments were performed in triplicate.

### 2.8 Chemotherapy drug sensitivity MTT assays

Drug sensitivity was determined using a 3‐(4,5‐dimethylthiazol‐2‐yl)‐2,5‐diphenyltetrazolium bromide (MTT; Sigma‐Aldrich) assay. To evaluate the reversal of drug resistance by miR, briefly, osteosarcoma KHOS_MR_ and U‐2OS_MR_ MDR cells were transfected with miR‐15b precursor as described above and plated in a 96‐well plate. Doxorubicin was added in appropriate concentrations 24 h later. An equivalent amount of diluent (dimethyl sulfoxide) was added to culture medium as a negative control. After 96 h of drug incubation, 20 μL of MTT (20 mg·mL^−1^) was added to each well. After incubation for an additional 4 h, 200 μL of isopropanol–HCl solution was added to each well to dissolve the intracellular formazan crystal products. Absorbance was determined using a 96‐well SpectraMax plate reader (Molecular Devices) at 560 and 650 nm (background). Drugs at the concentrations utilized in the MTT assay were performed in the absence of cells to verify no change in absorbance. Response curves were fitted using graphpad prism 4 software (GraphPad PRISM^®^ Software; GraphPad Software, San Diego, CA, USA).

### 2.9 Western blot analysis

Total protein from osteosarcoma cell lines was extracted by 1× RIPA lysis buffer (Upstate Biotechnology, Charlottesville, VA, USA). Protein concentration was determined by the DC Protein Assay (Bio‐Rad, Hercules, CA, USA). Equal amounts of protein were separated by NuPAGE^®^ 4–12% Bis‐Tris Gel (Life Technologies), transferred onto nitrocellulose membrane (Protran^®^; Whatman GmbH, Dassel, Germany) and incubated with Wee1‐specific primary antibodies (Santa Cruz Biotechnology Inc**.**, Cat# sc‐5285, Dallas, TX, USA, dilution 1 : 1000) and β‐actin (Sigma‐Aldrich, dilution 1 : 2000) at 4 °C overnight. The membranes were further probed with respective secondary antibodies (LI‐COR Biosciences, Lincoln, NE, USA) and scanned by Odyssey^®^ CLx imaging equipment (LI‐COR Biosciences) to detect the bands and the density. Densitometric analysis of western blot results was performed with ImageJ as described in the software's user guide. The relative expression of Wee1 was normalized with respect to actin expression. The western blot was performed in triplicate.

### 2.10 Luciferase assay

For luciferase (Luc) activity analysis, osteosarcoma KHOS_MR_ and U‐2OS_MR_ MDR cells were seeded in 96‐well plates, and 100 ng of Luc‐Wee1‐3′‐UTR reporter vector (SwitchGear Genomics, Menlo Park, CA, USA) and 40 nm of miR‐15 precursor or 40 nm of nonspecific miR precursor control vectors were cotransfected with Lipofectamine 3000 (Life Technologies). Luciferase activity was measured 48 h after transfection by the LightSwitch Luciferase Assay Reagent™ (SwitchGear Genomics).

### 2.11 Establishment of *in vivo* MDR osteosarcoma xenograft model and miR‐15b mimic treatment

To determine the effect of restoring miR‐15 expression on reversing drug resistance in osteosarcoma *in vivo*, approximately 2 × 10^6^ human osteosarcoma KHOS_MR_ MDR cells were resuspended in Matrigel (BD Biosciences, San Jose, CA, USA) in a 1 : 1 volume ratio and injected subcutaneously into the right hind flanks of 4‐ to 6‐week‐old Crl:SHO‐*Prkdc* ^SCID^ *Hr* ^hr^ nude mice (Charles River Laboratories, Ann Arbor, MI, USA). Two weeks after injection, when the average tumour volume reached ~ 100 mm^3^, the mice were randomized into three groups (*N* = 10 per group). Group 1 received injections with sterile saline (0.9% NaCl), group 2 with 2 mg·kg^−1^ doxorubicin and group 3 with the combination of *in vivo*‐ready mirVana™ miR‐15b mimics (Life Technologies) and doxorubicin (obtained from residual clinical materials provided by the pharmacy at the Massachusetts General Hospital). These *in vivo*‐ready miR mimics are nontoxic and did not induce an immune response in the animal models tested. miR‐15b mimics were injected via tail vein with Invivofectamine^®^ 2.0 Reagent following the manufacturer's instructions. Doxorubicin was injected intraperitoneally with a dose of 2 mg·kg^−1^ animal body weight. Two weeks after injection of KHOS_MR_ MDR cells, when the average tumour volume reached ~ 100 mm^3^, saline, doxorubicin alone or doxorubicin in combination with *in vivo*‐ready mirVana™ miR‐15b mimics was administered twice per week four times. For validation, we also included a group of mice with osteosarcoma KHOS_MR_ MDR cells previously transfected with miR‐15b precursor, established before subcutaneous injection. Effects on tumour growth rate were assessed in each mouse by determining the tumour volume on the day of treatment relative to the tumour volume on day 0. Tumour volume (mm^3^) was determined by digital calliper measurements and calculated as W^2^ × L/2, where W is width and L is length. Data are presented as mean ± SD. Animals were killed, once morbidity became evident or their tumour size exceeded 800 mm^3^. Following the indicated treatments, tumour tissues from the above treated animals were collected and placed in 10% formalin and embedded in paraffin for histological analysis. The effects of the miR‐15b mimic treatment on Wee1 expression were determined by immunohistochemical staining as described above. All animal studies were in compliance with the protocol approved by the Massachusetts General Hospital Subcommittee on Research Animal Care (SRAC) under the protocol number 2009N000229.

### 2.12 Osteosarcoma tissue microarray and immunohistochemistry

The osteosarcoma tissue microarray (TMA) has been described previously and contains 114 tumour tissues. The expression levels of Wee1 were determined following the immunohistochemistry protocol as previously reported (Ryu *et al*., 2010). A similar immunohistochemistry protocol was also used to determine Wee1 expression in osteosarcoma tissues from the mouse xenograft model. Wee1‐positive samples were defined as those showing nuclear staining pattern in the tumour tissues. Wee1 staining patterns were categorized into six groups: 0, no nuclear staining; 1+, < 10% of cells nuclear‐stained positive; 2+, 10–25% positive cells; 3+, 26–50% positive cells; 4+, 51–75% positive cells; 5+, > 75% positive cells. The percentage of cells showing positive nuclear staining for Wee1 was calculated by reviewing the entire spot. Categorization of Wee1 staining was completed by two independent investigators. Discrepant scores between the two investigators were rescored to obtain a single final score.

### 2.13 Statistical analyses

Kaplan–Meier survival analysis (GraphPad PRISM^®^) was used to analyse the correlation between the level of Wee1 expression and prognosis. Student's *t*‐test was used to compare the differences between groups. Results are given as mean ± SD, and values with *P* < 0.05 were considered statistically significant.

## 3 Results

### 3.1 Identification of differentially expressed miR‐15b in multidrug‐resistant osteosarcoma cell lines KHOS_MR_ and U‐2OS_MR_

**Figure 1:**
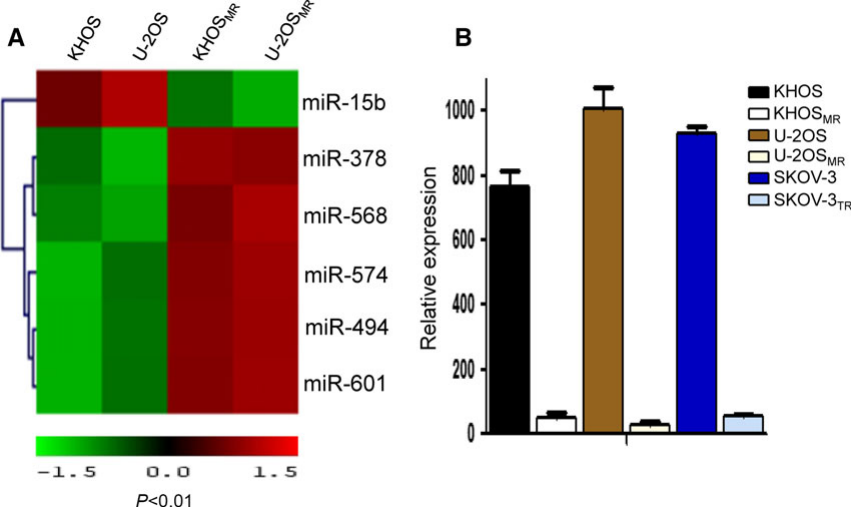
Expression of miR‐15b is decreased in multidrug‐resistant osteosarcoma cell lines. (A) Hierarchical clusters of most significantly (*P* < 0.01) altered miRs in drug‐sensitive (KHOS, U‐2OS) and drug‐resistant (KHOS_MR_, U‐2OS_MR_) osteosarcoma cell lines. miR array of MDR cell line pairs. Supervised hierarchical clustering of cell lines based on expression of miRs based on multiple‐sample analysis for each cell line. Each column represents a cell line, and each row represents a probe set. The heat maps indicate high (red) or low (green) level of expression relative to the mean as per the scale shown (the scale below is logarithmic). (B) Confirmation of decreased expression of miR‐15b in a panel of drug‐resistant cell lines (KHOS_R__2_, U‐2OS_MR_, SKOV‐3_TR_) as compared with their parental drug‐sensitive cell lines by TaqMan real‐time RT‐PCR. Bars represent levels of relative expression of miR‐15b in each cell line (*P* < 0.001).

To investigate the potential function of miRs in the development of drug resistance in osteosarcoma, global miR expression microarrays were used to analyse the miR expression profiles in the human osteosarcoma cell lines KHOS and U‐2OS, and their drug‐resistant variants KHOS_MR_ and U‐2OS_MR_. Cluster analysis revealed that KHOS and U‐2OS osteosarcoma cells with acquired resistance to doxorubicin were characterized by significant changes in miR expression. We identified six miR genes (one downregulated miR‐15b and five upregulated miRs) that were the most dramatically differentially expressed (*P* < 0.01) in KHOS_MR_ and U‐2OS_MR_ cells compared with their parental KHOS and U‐2OS cells (Fig. 1A). Among these differentially expressed miRs, miR‐15b, which has been recently shown to be implicated in a number of human cancers, was the only miR significantly (*P* < 0.01) downregulated in both KHOS_MR_ and U‐2OS_MR_ cell lines. As miR‐15b was most highly and concordantly downregulated in more than one osteosarcoma MDR cell line (both KHOS_MR_ and U‐2OS_MR_), we further investigated the expression of miR‐15b in the ovarian cancer MDR cell line SKOV‐3_TR_. The results of miR‐15b downregulation obtained by miR microarray analysis were independently confirmed by real‐time RT‐PCR in these MDR cell lines, suggesting that the association between miR‐15b and drug resistance is not cell line‐specific (Fig. 1B).

The mechanisms underlying miR deregulation in cancer are not well understood, and the results are controversial. Because many miRs have been aligned to genomic fragile sites or in cancer‐associated regions, genome copy abnormalities could involve miR genes. To determine whether miR‐15b copy numbers are associated with downregulation of miR‐15b expression in osteosarcoma MDR cell lines, we performed the miR‐15b TaqMan Copy Number Assay. The results showed that the osteosarcoma KHOS cell line had a higher copy number than the drug‐resistant KHOS_MR_ cell line (2.15 ± 0.07 and 1.75 ± 0.03, respectively). Human ovarian cancer cell line SKOV‐3 also had a higher copy number than drug‐resistant SKOV‐3_TR_ (2.05 ± 0.07 and 1.94 ± 0.06, respectively), suggesting that copy number changes of miR‐15b may at least partially contribute to miR‐15b downregulation in these MDR cells. However, osteosarcoma cell line U‐2OS had a lower copy number than drug‐resistant U‐2OS_MR_ (1.84 ± 0.11 and 2.19 ± 0.04, respectively) and human breast cancer cell line MCF‐7 also had a lower copy number than the drug‐resistant MCF‐7_TR_ (1.89 ± 0.11 and 2.02 ± 0.05, respectively) (Fig. S1). These data suggest that miR‐15b copy number variation at the genomic DNA level may not control the expression level of miR‐15b in all cell types, but additional regulatory mechanisms, including epigenetic changes, such as DNA methylation, or post‐transcriptional regulation may also be involved in miR‐15b deregulation. In addition, as many studies cannot identify a correlation between genomic DNA copy number and epigenetic changes and expression levels of many miRs, these data suggest that expression of miR may be regulated through different mechanisms than those that regulate expression of mRNA.

### 3.2 Restoration of miR‐15b results in the reversal of resistance of KHOS_MR_ and U‐2OS_MR_ cells to doxorubicin

**Figure 2:**
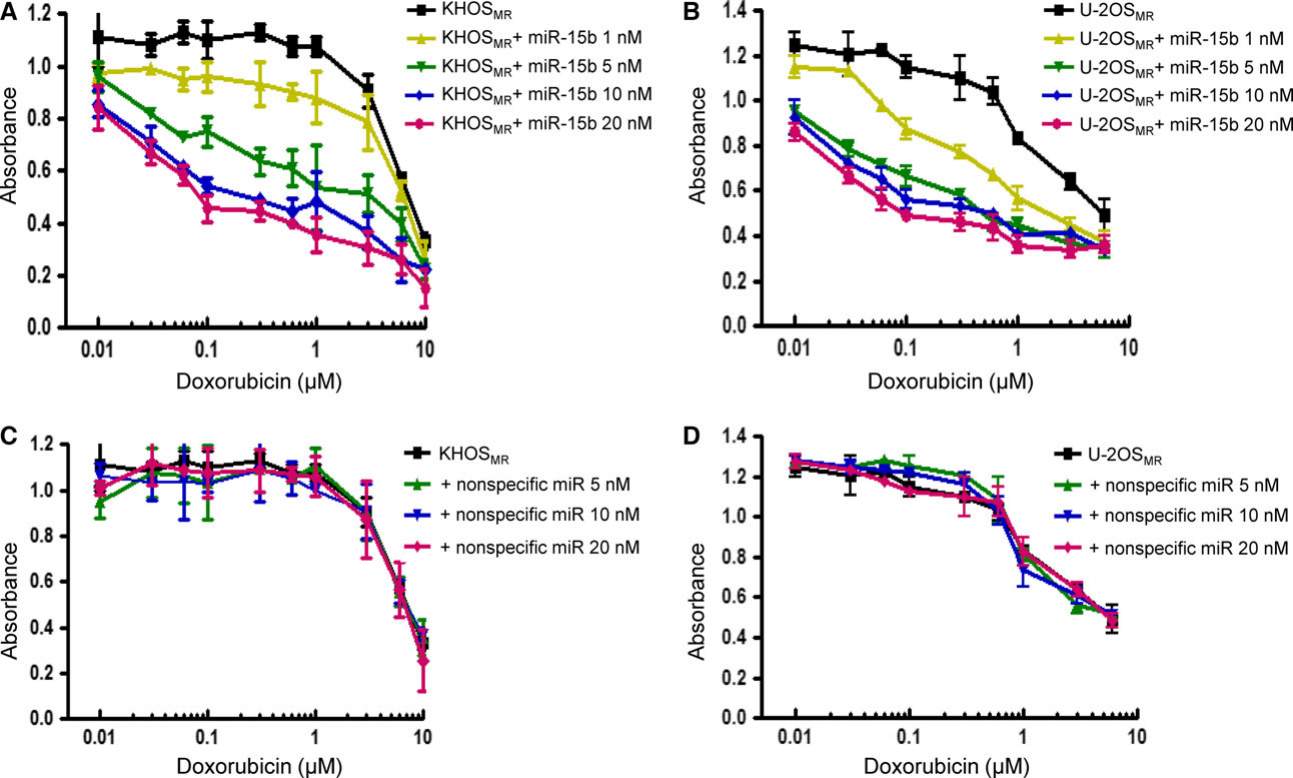
miR‐15b transfection into multidrug‐resistant osteosarcoma cells reverses doxorubicin resistance. KHOS_MR_ and U‐2OS_MR_ cells were transfected with either miR‐15b precursor or control nonspecific miR precursor and treated with different concentrations of doxorubicin 24 h after transfection. The relative sensitivity to doxorubicin was determined by MTT analysis 4 days post‐treatment. (A, B) Effect of miR‐15b expression on reversing drug resistance in KHOS_MR_ and U‐2OS_MR_ cell lines (*P* < 0.05). (C, D) No effect of nonspecific miR expression on reversing drug resistance in KHOS_MR_ and U‐2OS_MR_ cell lines. Values of absorbance reflect cell survival in miR‐15b‐transfected and doxorubicin‐treated cells.

The finding that miR‐15b expression was downregulated in both drug‐resistant osteosarcoma cell lines KHOS_MR_ and U‐2OS_MR_ suggests that loss of miR‐15b expression may contribute to cancer drug resistance; therefore, the restoration of miR‐15b expression in the resistant KHOS_MR_ and U‐2OS_MR_ cells may increase their sensitivity to doxorubicin. To test this hypothesis, we transfected KHOS_MR_ and U‐2OS_MR_ cell lines with miR‐15b precursor (with nonspecific miR precursor as control) and determined the sensitivity of cells to doxorubicin treatment. Figure 2A,B shows that transfection of KHOS_MR_ and U‐2OS_MR_ cells with miR‐15b precursor resulted in increased sensitivity of cells to doxorubicin in a dose‐dependent manner, whereas transfection with control nonspecific miR precursor had no effect on doxorubicin sensitivity in both cell lines (Fig. 2C,D).

### 3.3 Wee1 is a direct target of miR‐15b in osteosarcoma cells

**Figure 3:**
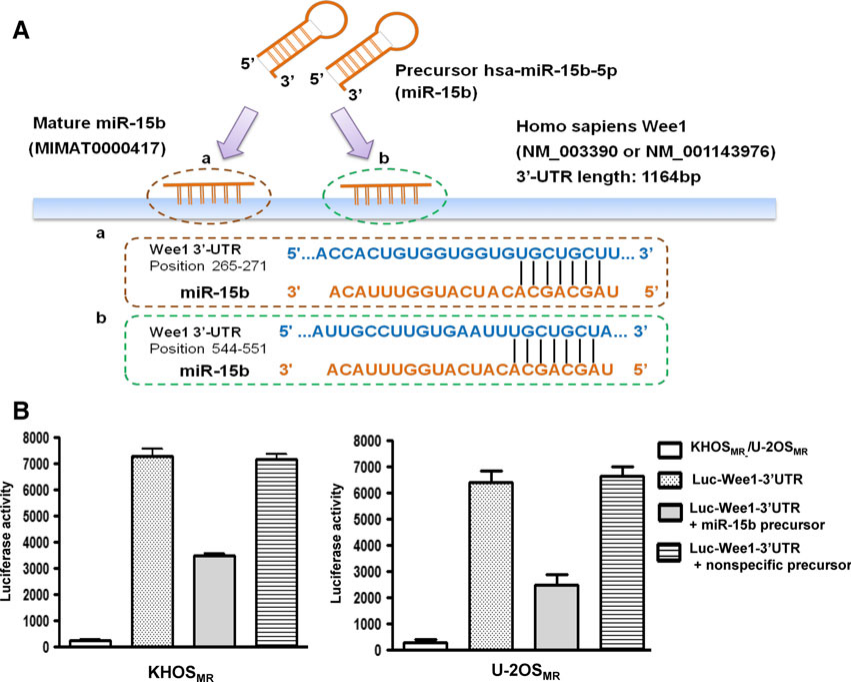
Wee1 is a direct target of miR‐15b in osteosarcoma MDR cells. (A) Computational analysis of miR databases identified Wee1 as a potential target of miR‐15b. Two binding sites of miR‐15b exist in the 3′‐UTR of human Wee1 gene mRNA. (B) Effect of miR‐15b expression on Wee1 driven luciferase activity. KHOS_MR_ and U‐2OS_MR_ cells were transfected with a luciferase reporter construct fused to the Wee1 3′‐UTR. Subsequently, cells were cotransfected with miR‐15b precursor or control nonspecific miR precursor. Luciferase activities were measured 48 h after transfection using the LightSwitch Luciferase Assay Reagent^™^. Values are shown as the percentage of luciferase expression compared with the controls (*P* < 0.01).

Mature miRs bind to the 3′‐untranslated regions (3′‐UTRs) of target genes and inhibit gene expression by degradation or repression of translation of the target mRNA. To identify the miR‐15b target gene that may play a role in drug resistance in osteosarcoma, we employed a number of available computational analysis databases of miR, including microRNA.org and TargetScan databases, to search for putative binding sites of miR‐15b in the 3′‐UTR of human gene mRNAs. One of the top‐ranked and highest scoring gene mRNAs was the Wee1 gene. Further analysis showed that the predicted alignment of miR‐15b targeted two specific sequence sites in the Wee1 mRNA at nucleotide 265–271 and 544–551 from the start of the Wee1 3′‐UTR of 1164 base pairs (Fig. 3A). This prediction was further confirmed by the mirgen software. Therefore, Wee1 could be a potential direct target of miR‐15b. To examine whether Wee1 is indeed functionally targeted by miR‐15b, the segment of Wee1 3′‐UTR containing the miR‐15b complementary site was cloned into the 3′‐UTR of a luciferase reporter system. This reporter system was designed to validate predicted miR targets in the 3′‐UTR of a specific gene. The resulting Wee1 reporter vector was cotransfected into KHOS_MR_ and U‐2OS_MR_ cells with the miR‐15b precursor or the nonspecific miR precursor that does not have binding sites within the 3′‐UTR of Wee1. Figure 3B shows that miR‐15b inhibited the luciferase activity from the construct with the Wee1 3′‐UTR segment in both KHOS_MR_ and U‐2OS_MR_ cells. There was no change in the luciferase reporter activity when the cells were cotransfected with the negative control nonspecific miR precursor (Fig. 3B) or unrelated miRs, such as miR‐1 and miR‐199a‐3p (data not shown).

### 3.4 miR‐15b significantly inhibits Wee1 expression

**Figure 4:**
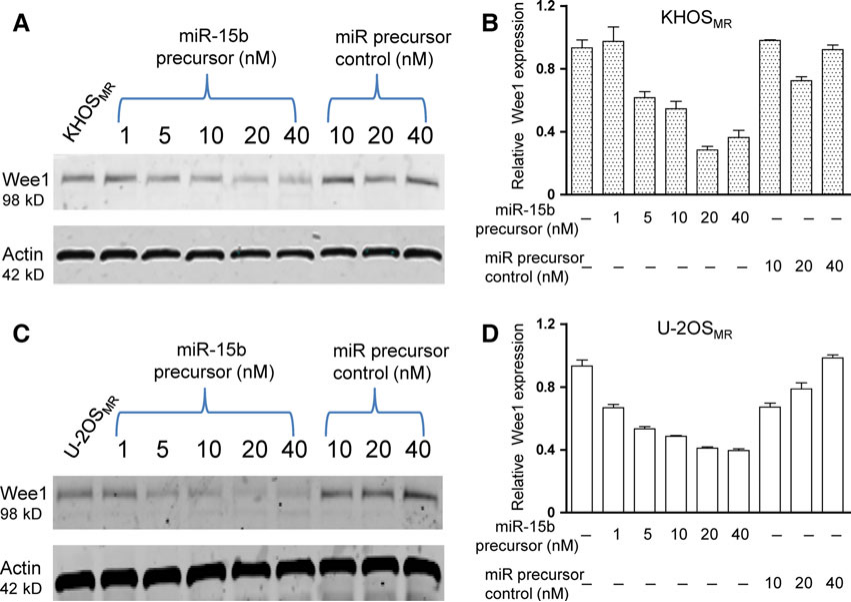
miR‐15b transfection into multidrug‐resistant osteosarcoma cells suppresses Wee1 expression. (A, C) miR‐15b transfection into osteosarcoma drug‐resistant KHOS_MR_ and U‐2OS_MR_ cells suppresses Wee1 expression. KHOS_MR_ and U‐2OS_MR_ cells were transfected with either miR‐15b precursor or control nonspecific miR precursor in a dose‐dependent manner. Expression Wee1 was determined by western blot 48 h after transfection. β‐Actin was used as an endogenous control. (B, D) Western blots from A and C were analysed by densitometry with ImageJ as described in the Materials and Methods. The relative expression of Wee1 was normalized with respect to actin expression. The western blot was performed in triplicate as indicated by the error bars. miR‐15b significantly inhibits Wee1 expression compared with controls (*P* < 0.05).

To further confirm that miR‐15b indeed affects the protein levels of Wee1 in KHOS_MR_ and U‐2OS_MR_ cells, these cells were transfected with either the miR‐15b precursor (1, 5, 10, 20, or 40 nm) or control nonspecific miR precursor (10, 20 or 40 nm), and the level of Wee1 was determined by western blot 48 h after transfection. The results showed that transfection of KHOS_MR_ and U‐2OS_MR_ cells with miR‐15b resulted in a decrease in Wee1 levels, whereas the transfection with the control nonspecific miR precursor had no effects on the expression of Wee1 (Fig. 4A,C). Quantitative analysis of the western blot data showed that transfection with miR‐15b reduced Wee1 expression in a dose‐dependent manner in both KHOS_MR_ (Fig. 4B) and U‐2OS_MR_ cells (Fig. 4D). In both of these Pgp‐overexpressed KHOS_MR_ and U‐2OS_MR_ MDR osteosarcoma cell lines, transfection with miR‐15b had no effect on Pgp expression (data were not shown).

### 3.5 Combination of miR‐15b and doxorubicin treatment reverses drug resistance

**Figure 5:**
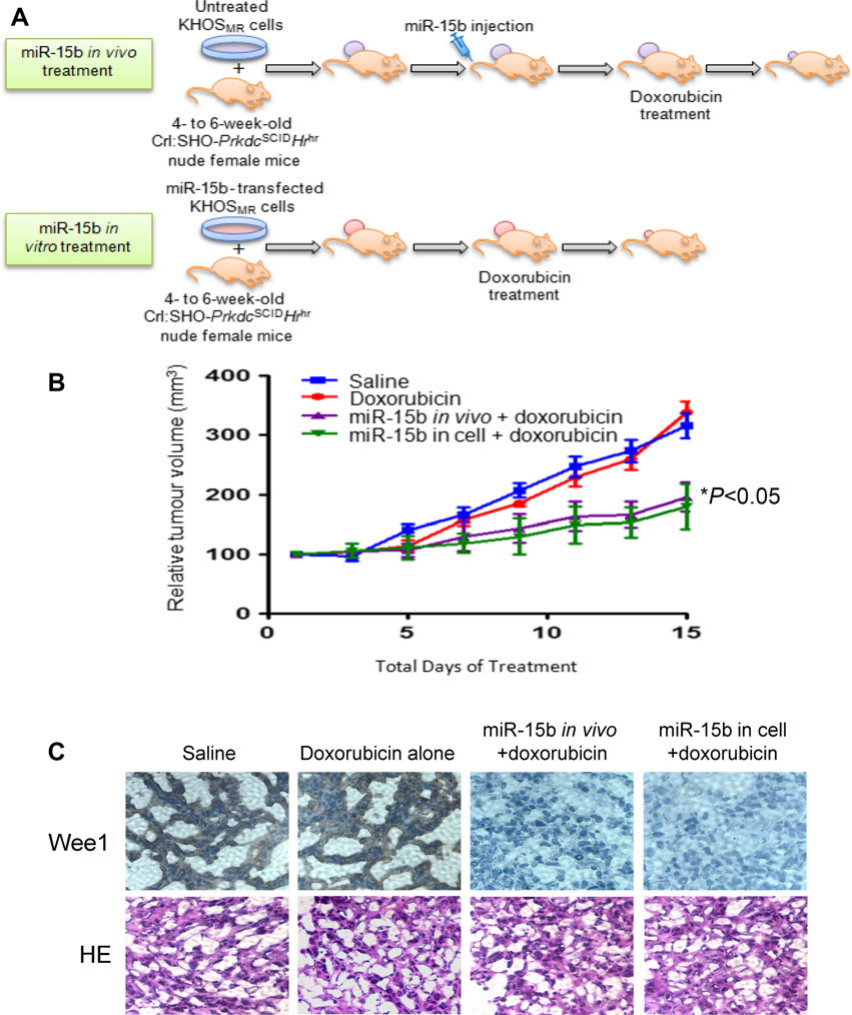
Reversal of doxorubicin resistance in osteosarcoma KHOS_MR_ MDR cells *in vivo*. (A) A suspension of KHOS_MR_ cells was injected subcutaneously into the flank of a nude mouse. Two weeks after injection, when the average tumour volume reached ~ 100 mm^3^, saline, doxorubicin (2 mg·kg^−1^, injected intraperitoneally) alone or doxorubicin in combination with *in vivo*‐ready mirVana^™^ miR‐15b mimics (injected via tail vein) were administered twice per week four times. (B) Effects on tumour growth rate were assessed in each mouse by determining the tumour volume on the day of treatment relative to the tumour volume on day 0. Tumours treated with saline are shown in blue, tumours treated with doxorubicin alone in red, and tumours treated with a combination of miR‐15b mimics and doxorubicin in green (KHOS_MR_ cells was directly injected subcutaneously and then treated with *in vivo*‐ready mirVana^™^ miR‐15b mimics by tail vein injection). KHOS_MR_ tumours previously transfected with miR‐15b precursor before subcutaneous injection are shown in pink. Curves for representative tumour volume per group (*N* = 10) and tumour volume were presented as mean ± SD (*P < 0.05). (C) Histological analysis of the effect of miR‐15b mimics on Wee1 staining in osteosarcoma tumour tissues shows downregulation of Wee1 expression compared with saline or treatment with doxorubicin alone.

To further support the biological significance of the miR‐15b findings in MDR osteosarcoma *in vitro*, we subsequently investigated the role of miR‐15b in reversing drug resistance *in vivo*. The MDR osteosarcoma cells KHOS_MR_ were either directly grown as xenografts in nude mice or transfected with miR‐15b precursor prior to subcutaneous injection. The treatment scheme is shown in Fig. 5A. The results revealed that treatment with saline alone or doxorubicin alone had no obvious beneficial effects on MDR osteosarcoma tumour growth suppression in xenograft models. In contrast, treatment with the combination of miR‐15b and doxorubicin produced a significant inhibitory effect on the growth of the resistant tumours as compared with control groups (Fig. 5B). The immunohistochemical staining indicated a significant decrease in Wee1 expression in tumours treated with *in vivo*‐ready mirVana™ miR‐15b mimics and in tumours established with KHOS_MR_ cells previously transfected with miR‐15b precursor (Fig. 5C). Additionally, on the basis of animal weight and mortality, no considerable toxicity was observed and the animals appeared to have tolerated miR‐15b well in all the treatment regimens (Fig. S2).

### 3.6 Wee1 knockdown increases the cytotoxic effect of doxorubicin

Downregulation of miR‐15b‐associated Wee1 expression may participate in the drug resistance in osteosarcoma. We hypothesized that direct inhibition of the Wee1 pathway in osteosarcoma cells may lower the apoptotic threshold and increase chemotherapy sensitivity. To determine whether Wee1 downregulation influenced the effect of the chemotherapeutic drug doxorubicin in osteosarcoma cells, KHOS_MR_ and U‐2OS_MR_ cells were transfected with Wee1 siRNA and then incubated with doxorubicin, a commonly used first‐line chemotherapy drug in the treatment of osteosarcoma. The combination of Wee1 siRNA and doxorubicin significantly inhibited cell growth and survival as compared with those from each treatment alone in KHOS_MR_ or U‐2OS_MR_ cell lines (Fig. S3). These results showed that the Wee1 knockdown enhanced the cytotoxic effect of doxorubicin in osteosarcoma MDR cells.

### 3.7 miR‐15b expression levels correlate with clinical prognosis in patients with osteosarcoma

**Figure 6:**
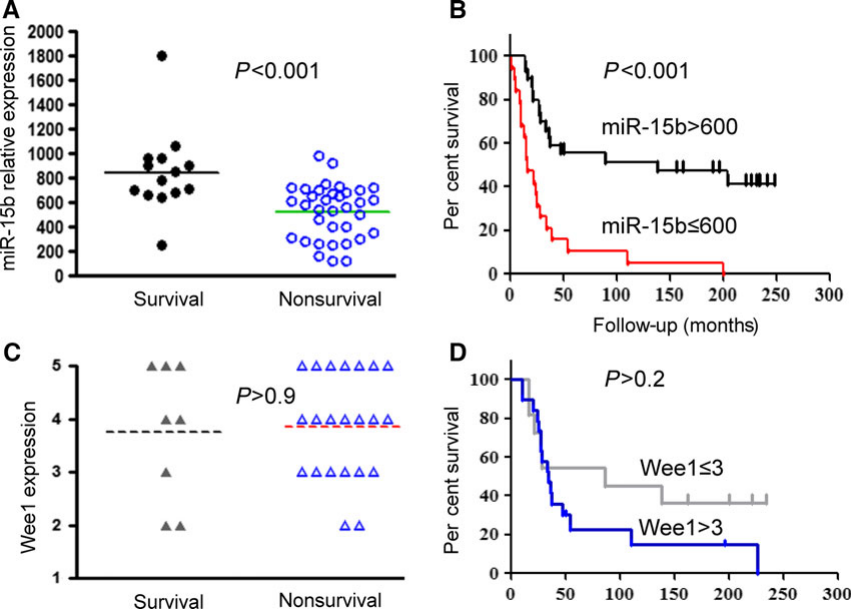
Correlation of miR‐15b and Wee1 with survival of patients with osteosarcoma. (A) Distribution of miR‐15b expression in survival and nonsurvival of osteosarcoma patient samples as determined by real‐time RT‐PCR (*P* < 0.001). (B) Analyses of association between expression of miR‐15b (miR‐15b low level ≤ 600 and miR‐15b high level > 600) and survival in patients with osteosarcoma (*P* < 0.001). (C) Distribution of Wee1 expression in survival and nonsurvival of osteosarcoma patient samples as determined by immunohistochemistry (*P* > 0.9). (D) Association between expression of Wee1 (Met level ≤ 3+ and Wee1 level > 2+) and survival in patients with osteosarcoma (*P* > 0.2). Kaplan–Meier survival analysis was used to analyse the correlation between the level of miR‐15b or Wee1 expression and survival.

To further validate the clinical relevance of miR‐15b expression in patients with osteosarcoma, we determined miR‐15b expression levels and analysed the correlation by a two‐sided Student's *t*‐test to compare the differences between the survival and nonsurvival groups of patients with osteosarcoma. A retrospective study of 49 patients with osteosarcoma from the MGH sarcoma tissue bank and orthopaedic oncology databases was surveyed for miR‐15b expression analysis. The data on each patient's age, gender, histological subtype, metastasis, recurrence, tumour location(s), follow‐up months and disease status were collected. The expression of miR‐15b in the 49 osteosarcoma samples was then determined by the TaqMan miR assay. A total of 14 (28.6%) samples from survivors and 35 (71.4%) samples from nonsurvivors were collected. Comparison of miR‐15b expression between the two groups of patients revealed that miR‐15b expression for samples from nonsurvivors was significantly lower than that of survivors (*P* < 0.001). The average miR‐15b expression levels for survivors and nonsurvivors were 846 and 526, respectively (Fig. 6A). By comparing the clinical characteristics of miR‐15b‐low (≤ 600) and miR‐15b‐high (> 600) groups of patients with osteosarcoma, Kaplan–Meier survival analysis showed that the outcome for patients in the miR‐15b‐low group was significantly worse than for those in the miR‐15b‐high group (Fig. 6B). Because miR‐15b inversely regulates Wee1 expression, we also evaluated whether downregulation of miR‐15b in osteosarcoma samples was correlated with Wee1 upregulation. In total, 27 frozen osteosarcoma samples with miR‐15b expression were evaluated against Wee1 expression from matched TMA samples for this analysis. The resulting correlation coefficient and *P*‐value of *r* = −0.28 and *P* < 0.005 indicated that miR‐15b expression was weakly but inversely correlated with Wee1 expression in osteosarcoma tissues (Fig. S4). We further analysed the correlation between Wee1 expression and the prognosis of patients with osteosarcoma in the osteosarcoma TMA samples. No significant difference in Wee1 expression was found between the survivor and nonsurvivor groups (Fig. 6C). The results also revealed that although there is a trend of higher (> 3+) Wee1 expression in patients with short‐term survival than lower (≤ 3+) Wee1 expression in patients with long‐term survival, no statistically significant difference (*P* > 0.2) in Wee1 expression was identified between these groups of patients (Fig. 6D).

## 4 Discussion

Drug resistance is a major cause of cancer progression during and after treatment with chemotherapy. miRs have been implicated in the development of drug resistance in various types of human cancers. However, little is known about the role of miRs in osteosarcoma drug resistance. In this study, we used drug‐resistant osteosarcoma MDR cell lines and their parental drug‐sensitive cell lines to investigate the molecular mechanism of resistance and the associated miR changes. We demonstrate that significantly reduced miR‐15b expression was associated with chemotherapeutic resistance in osteosarcoma MDR cell lines. miR‐15b was the most significantly downregulated miR identified in drug‐resistant osteosarcoma cell lines by global miR expression profiles analysis. Although the present study is solely focused on miR‐15b, other upregulated miRs that were identified in the initial screening of the osteosarcoma MDR cell lines, including miR‐378, may also be involved in drug resistance. Notably, overexpression of miR‐378 has been shown to enhance both cell survival and colony formation and contributed to drug resistance in glioblastoma cancer cells (Wu *et al*., 2012). Expression of miR‐574 has also been shown to be associated with chemoresistance in different cancers (Ranade *et al*., 2010; Ujihira *et al*., 2015).

Several lines of evidence consistently demonstrate that miR‐15b is critical for tumour cell growth, invasion/metastasis, angiogenesis and apoptosis (Lovat *et al*., 2015; Xia *et al*., 2008; Zhao *et al*., 2016). miR‐15b transfection into tumour cells can decrease tumour cell proliferation and induce apoptosis (Cimmino *et al*., 2005; Xia *et al*., 2008; Zheng *et al*., 2013). However, there are no reports on the use of delivery of miR‐15b *in vitro* and *in vivo* for reversing drug resistance for anticancer therapy. Our study demonstrated that restoration of miR‐15b expression is able to reverse drug resistance in osteosarcoma cells. Reduced expression of miR‐15b conferred drug resistance traits in part through the modulation of Wee1, which was validated by target prediction programs and experimentally *in vitro* and *in vivo*. We verified that miR‐15b expression negatively correlated with Wee1 levels in osteosarcoma cells. Ectopic expression of miR‐15b in osteosarcoma MDR cells reduced the expression of Wee1, which may contribute to drug resistance. However, transfection with miR‐15b had no effect on the expression of Pgp in the osteosarcoma MDR cell lines, suggesting that Wee1 or other Pgp‐independent mechanisms may be responsible for the miR‐15b‐associated drug sensitivities. Wee1 is a kinase that affects the G2‐M checkpoint in the cell cycle (Do *et al*., 2013). Inhibition of Wee1 has been shown to increase the antitumour activities in the chemotherapy agents that cause DNA damage (Kreahling *et al*., 2012). Several more recent studies have shown that Wee1 inhibition overcomes drug resistance in different cancers (Aarts *et al*., 2015; Hamilton *et al*., 2014; Osman *et al*., 2015).

Previous study has shown that miR‐15b expression suppressed metastasis of drug‐resistant tongue cancer cells in mouse xenograft model (Sun *et al*., 2012). In our study, the effect of miR‐15b on reversing drug resistance *in vivo* in a mouse xenograft model of drug‐resistant osteosarcoma was measured. Without miR‐15b treatment, doxorubicin alone was unable to suppress drug‐resistant osteosarcoma tumour growth. However, treatment with the combination of miR‐15b and doxorubicin significantly delayed tumour growth *in vivo*. Importantly, immunohistochemistry analyses of these tumours showed decreased levels of Wee1 expression *in vivo*. Thus, these data suggest that miR‐15b expression is not only a potential prognostic marker, but also a possible therapeutic target in osteosarcoma.

The development of drug resistance is a major cause of cancer recurrence in osteosarcoma. A relapse phenotype may be acquired *via* chemotherapy‐induced selection of a resistant subpopulation or adaptation of the original tumour cells to therapy drugs. High levels of miR‐15b expression have been found associated with low risk of recurrence in several types of cancer (Chung *et al*., 2010; Sun *et al*., 2012). Increased expression of miR‐15b in pretreatment osteosarcoma samples has been demonstrated as the most stringent predictor of good response to chemotherapy, in which higher expression of miR‐15b in pretreatment samples correlated with subsequent positive response to chemotherapy (Jones *et al*., 2012). Expression levels of miR‐15b in pretreatment specimens also correlate positively with percentage of necrosis following neoadjuvant chemotherapy (Jones *et al*., 2012). In this study, we evaluated whether there is a correlation between the level of miR‐15b expression and clinical outcome in osteosarcoma. Comparison of miR‐15b expression showed that miR‐15b expression for samples from nonsurvivors was significantly lower than that of survivors in osteosarcoma. The outcome for patients in the miR‐15b‐low group was significantly worse than for those in the miR‐15b‐high group. The data confirmed that miR‐15b expression levels also correlated with clinical prognosis, as low expression of miR‐15b is a predictor of poor prognosis in patients. These results are consistent with previous reports in that miR‐15b levels were found to be significantly lower in shorter‐survival groups as compared with longer‐survival groups of patients with tongue cancer or liver cancer (Hattinger *et al*., 2015; Sun *et al*., 2012).

Several studies have demonstrated that miR‐15b and its downstream targeted genes are dysregulated in human cancer cells, including in drug‐resistant cancer cells (Allen and Weiss, 2010; Cimmino *et al*., 2005; Migliore *et al*., 2008). miR‐15b transfection into cancer cells has been shown to lead to decreased expression of Bcl‐2, cyclin E and PIM1 proteins, providing additional mechanistic explanations of how miR‐15b might play an important role in the development drug resistance (Migliore *et al*., 2008; Weirauch *et al*., 2013; Xia *et al*., 2009). Bcl‐2 causes chemoresistance in sarcoma, and chemosensitivity to doxorubicin and cisplatin can be restored by the Bcl‐2 inhibitor ABT‐737 (van Oosterwijk *et al*., 2012). The decrease or loss of miR‐15b expression and the overexpression of Wee1 have been related to invasive growth or tumour stages in several human cancers (Allen and Weiss, 2010; Chung *et al*., 2010; Cimmino *et al*., 2005; Kreahling *et al*., 2012; PosthumaDeBoer *et al*., 2011; Ryu *et al*., 2010; Weirauch *et al*., 2013). However, the dynamics and integrity of miR‐15b in relation to evolving drug resistance in osteosarcoma in clinical settings have not been investigated. We determined the correlation between expression of miR‐15b and Wee1 in osteosarcoma tissues. The results showed that miR‐15b expression was inversely correlated with Wee1 expression; expression of miR‐15b was lower in osteosarcoma tissues and was coupled with high expression of Wee1. These results are consistent with studies in other types of human cancers, such as colorectal tumours, which also show that miR‐15b and Wee1 are often concomitantly deregulated (Zhao *et al*., 2016). Our study also showed that miR‐15b expression correlated with clinical prognosis in patients with osteosarcoma; the outcome for patients in the miR‐15b‐low group was significantly worse than for those in the miR‐15b‐high group. For Wee1 expression, although there was a trend of higher Wee1 expression in patients associated with short‐term survival than in patients associated with long‐term survival, no statistically significant difference in Wee1 expression was found between these groups of patients. The discrepancy between miR‐15b and Wee1 expression for clinical prognosis in osteosarcoma may relate to multiple potential target genes of miR‐15b. In theory, each miR is believed to interact with up to 200 potential target genes; a single miR can regulate multiple oncogenes and oncogenic pathways that are commonly deregulated in different cancers. As such, miR‐15b has been reported to regulate Wee1, Bcl‐2, cyclin E, cyclin D and/or IGF‐1R expression in several types of cancer, and many of these genes also play important roles in drug resistance and apoptosis resistance (Carthew and Sontheimer, 2009; van Oosterwijk *et al*., 2012; PosthumaDeBoer *et al*., 2011; Wu *et al*., 2012; Xia *et al*., 2009). However, the potential distinct profile of miR‐15b‐targeted genes in osteosarcoma is unknown and further studies are warranted. More recently, deletion of miR‐15b in knockout mice has been shown to promote B‐cell malignancies. miR‐15b/miR‐16‐2 knockout mice developed B‐cell malignancy by age 15–18 months with a penetrance of 60%. miR‐15b/miR‐16‐2 modulates the cyclin D1, cyclin D2 and IGF‐1R genes involved in proliferation and anti‐apoptotic pathways in mouse B cells (Wu *et al*., 2012). These data suggest that miR‐15b also plays an important role in the pathogenesis of B‐cell chronic lymphocytic leukaemia.

The G2‐M DNA damage checkpoint is an important cell‐cycle checkpoint in eukaryotic organisms ensuring that cells do not initiate mitosis before they repair damaged DNA after replication. miR‐15b‐targeted Wee1 is a tyrosine kinase with a key role as an inhibitory regulator of the G2‐M checkpoint that precedes entry into mitosis. There is compelling evidence that abrogation of this checkpoint through Wee1 inhibition may result in increased antitumour activity of agents that cause DNA damage, such as radiation therapy or some cytotoxic agents. For example, inhibition of Wee1 by the selective small‐molecule inhibitor MK1775 can abrogate the G2‐M checkpoint, resulting in premature mitotic entry and initiation of apoptotic cell death in all sarcoma cells tested. The cytotoxic effect of Wee1 inhibition on sarcoma cells seems to be p53 status‐independent as all sarcoma cell lines with different p53 mutations were highly sensitive to MK1775 treatment. MK1775 significantly enhanced the cytotoxic effect of gemcitabine in sarcoma cells lines with different p53 mutational status (Kreahling *et al*., 2013). Wee1 inhibition is also effective in patient‐derived sarcoma cells; MK1775 as a single agent causes significant apoptotic cell death, suggesting that Wee1 inhibition may represent a novel approach in the treatment of sarcomas. Wee1 inhibition has also been shown to sensitize osteosarcoma to radiotherapy (PosthumaDeBoer *et al*., 2011).

In conclusion, we demonstrate that miR‐15b is involved in drug resistance in osteosarcoma. We applied both *in vitro* and *in vivo* experimental and clinical findings to observe that miR‐15b is reduced in drug‐resistant osteosarcoma cells and tissues, which affected the chemosensitivity of osteosarcoma cells maybe partly through the regulation of Wee1. As miR‐15b and its targeted genes, including Wee1 and Bcl‐2, hold great potential for the treatment of chemoresistance, this research provides strong evidence for future development of miR‐based therapeutics strategies directed at treating osteosarcoma. miR‐15b‐ or miR‐15b‐targeted gene protein inhibitors may have a greater therapeutic potential in the treatment of drug‐resistant osteosarcoma when co‐administered with doxorubicin.

## Author contributions

ZD and FH conceived and designed the project. ZD, YG and JS performed the experiments. ZD, HM and FH provided the essential tools and reagents. ZD, YG, JS and FH analysed the data. ZD wrote the manuscript. ZD, JS, EC, GC, DH, KB, SLC and FH contributed to the interpretation and discussion of the results and reviewed the manuscript.

## Conflict of interest

E. Choy is a consultant or a member of advisory board for Amgen, Sanofi‐Aventis and BioMed Valley Discoveries, Inc. The other authors have declared no conflict of interest.

## Supporting information


**Fig. S1.** Relative abundance of miR‐15b genomic DNA copy numbers in drug sensitive and resistant cell lines.
**Fig. S2.** Administration of miR‐15b followed by doxorubicin treatment had no obvious effects on weight of mice in different groups of osteosarcoma xenograft model.
**Fig. S3.** Wee1 knockdown increases the cytotoxic effect of doxorubicin in KHOS_MR_ and U‐2OS_MR_ lines.
**Fig. S4.** miR‐15b expression in osteosarcoma samples was inversely correlated with Wee1 protein expression.
