# Supplementary material for: miR‐15b modulates multidrug resistance in human osteosarcoma in vitro and in vivo
Source: Mol Oncol. 2016 Oct 24;11(2):151–66. doi: 10.1002/1878-0261.12015 (PMC5300234; doi:10.1002/1878-0261.12015)

Supplementary Figure. S1

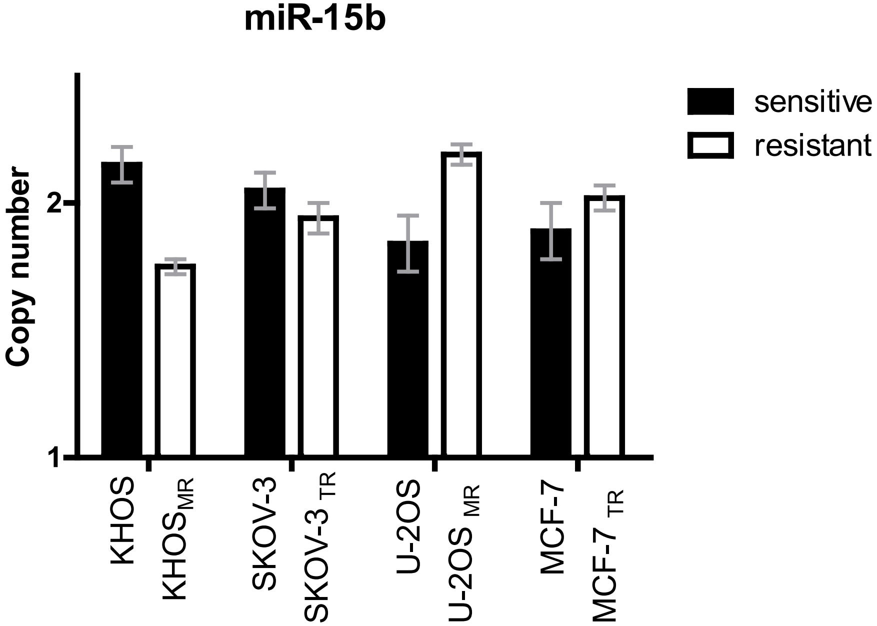

Supplementary Figure. S2

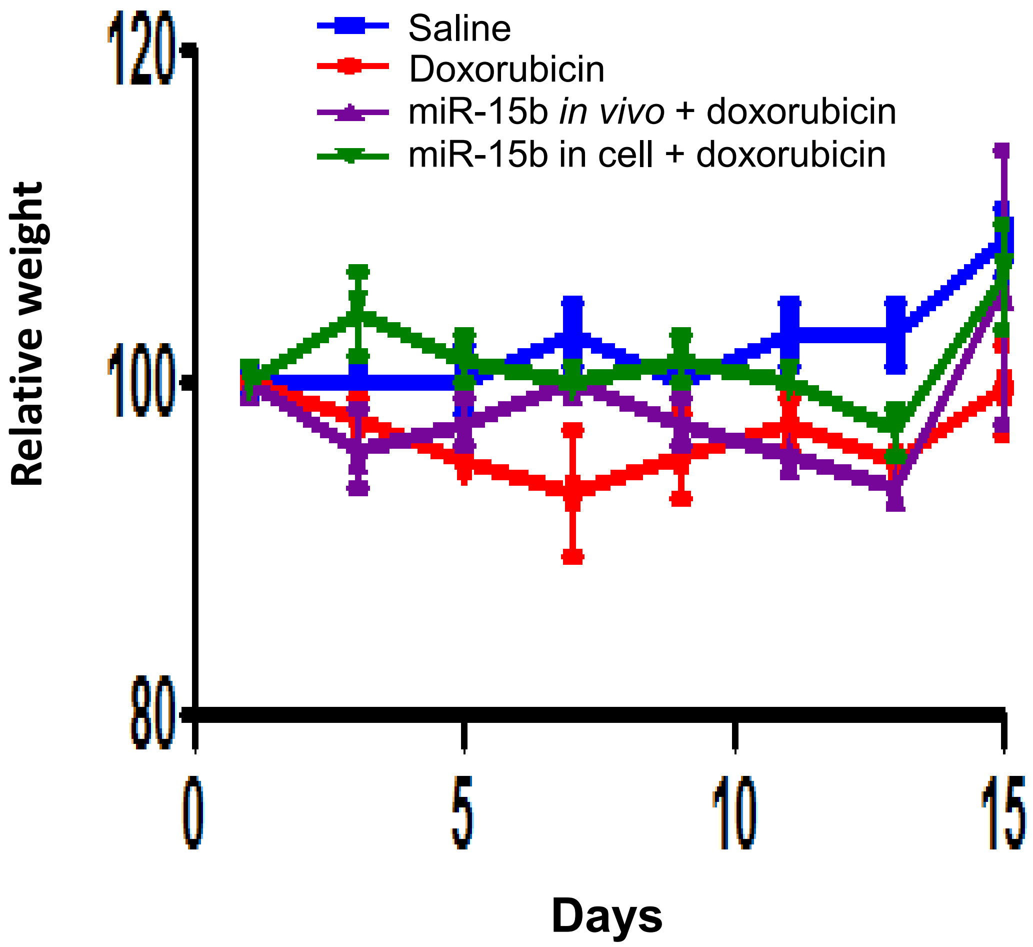

Supplementary Figure. S3

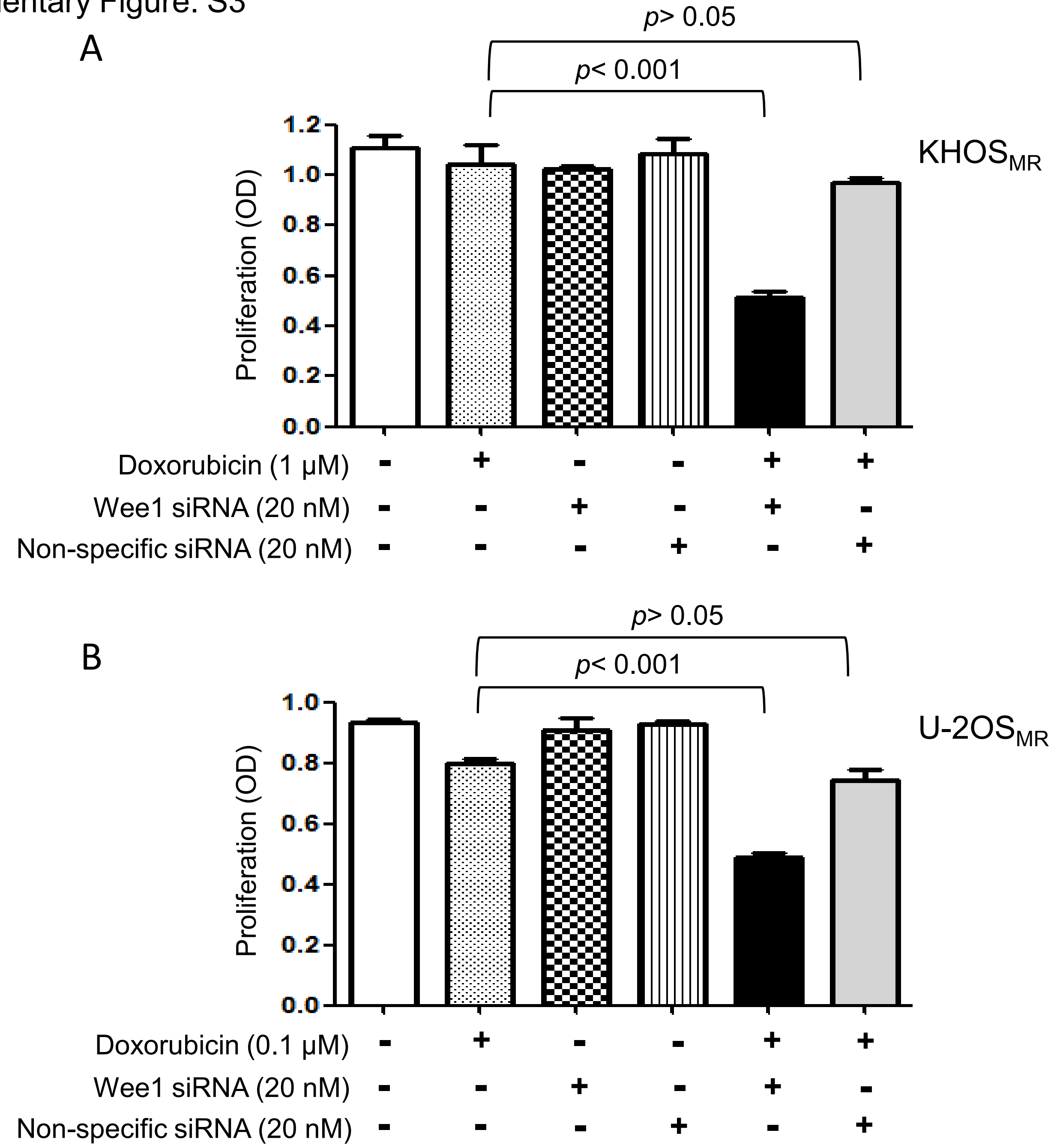

Supplementary Figure. S4

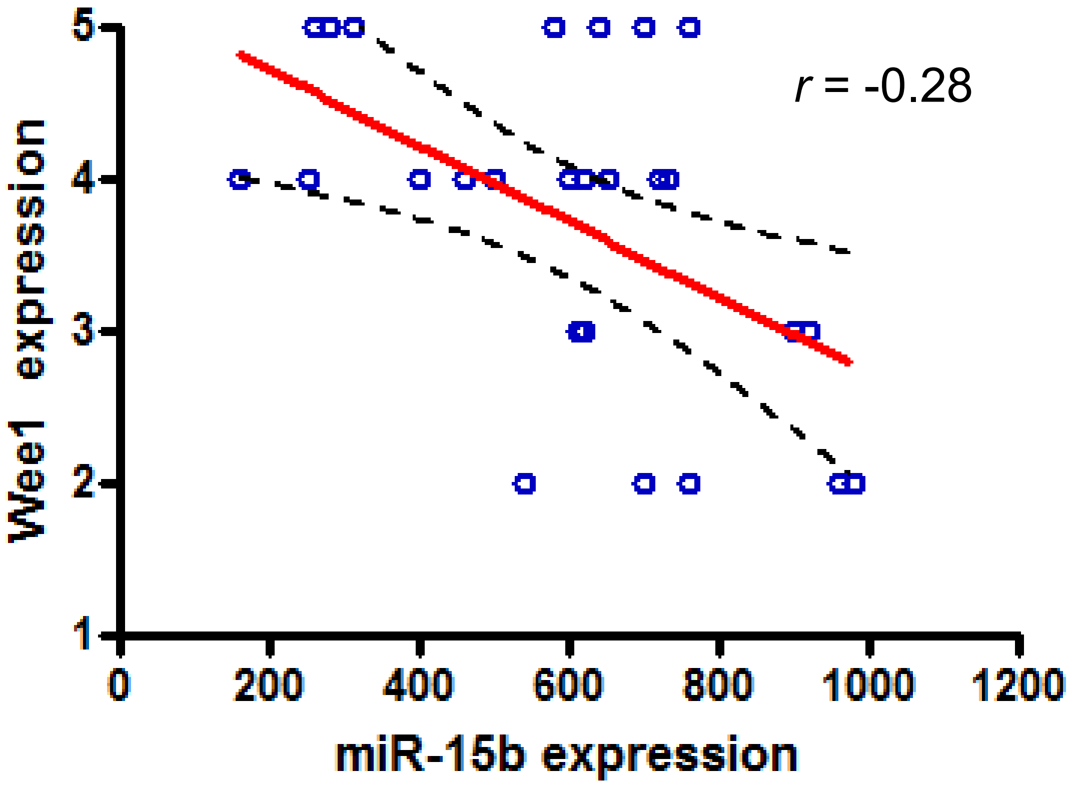

Supplement: Supplementary file 1 — Fig. S1. Relative abundance of miR‐15b genomic DNA copy numbers in drug sensitive and resistant cell lines. Fig. S2. Administration of miR‐15b followed by doxorubicin treatment had no obvious effects on weight of mice in different groups of osteosarcoma xenograft model. Fig. S3. Wee1 knockdown increases the cytotoxic effect of doxorubicin in KHOSMR and U‐2OSMR lines. Fig. S4. miR‐15b expression in osteosarcoma samples was inversely correlated with Wee1 protein expression. [file MOL2-11-151-s001.pdf]
