# Supplementary material for: miR‐15b modulates multidrug resistance in human osteosarcoma in vitro and in vivo
Source: Mol Oncol. 2016 Oct 24;11(2):151–66. doi: 10.1002/1878-0261.12015 (PMC5300234; doi:10.1002/1878-0261.12015)
Supplement: Supplementary file 2 [file MOL2-11-151-s002.docx]

**Supplementary Figure Legends**

**Supplementary Figure S1.** Relative abundance of miR-15b genomic DNA copy numbers in drug sensitive and resistant cell lines. To determine potential miR-15b copy number variation between chemoresistance and their parental sensitive cell lines, the TaqMan Copy Number Assay was performed. Results were analyzed on CopyCaller™ Software v2.0 with Manual C_T_ threshold: 0.2; Autobaseline: On; and Predicted Copy Number: 2. Data were presented as Copy Number ± SD.

**Supplementary Figure S2.** Administration of miR-15b followed by doxorubicin treatment had no obvious effects on weight of mice in different groups of osteosarcoma xenograft model. The weight of the mice was measured twice per week throughout the experiment.

**Supplementary Figure S3.** Wee1 knockdown increases the cytotoxic effect of doxorubicin in KHOS_MR_ and U-2OS_MR_ lines. The 20 nmol dose of Wee1 siRNA was chosen for KHOS_MR_ and U-2OS_MR_, following incubated with 1 and 0.1 µmol doses of doxorubicin for KHOS_MR_ and U-2OS_MR_, respectively. Proliferation was assessed by MTT as described in Materials and Methods. The data represent the mean ± SD of three experiments carried out in triplicate.

**Supplementary Figure S4.** miR-15b expression in osteosarcoma samples was inversely

correlated with Wee1 protein expression. Expression of miR-1 was evaluated by miR real-time PCR and expression of Wee1 was determined by immunohistochemistry. The correlation coefficient of r=-0.28 with p<0.005 indicates that miR-15b expression was inversely correlated with that of Wee1 expression.
